# Relevance of Platinum Underlayer Crystal Quality for the Microstructure and Magnetic Properties of the Heterostructures YbFeO_3_/Pt/YSZ(111)

**DOI:** 10.3390/nano14121041

**Published:** 2024-06-17

**Authors:** Sondes Bauer, Berkin Nergis, Xiaowei Jin, Lukáš Horák, Reinhard Schneider, Václav Holý, Klaus Seemann, Tilo Baumbach, Sven Ulrich

**Affiliations:** 1 Institute for Photon Science and Synchrotron Radiation, Karlsruhe Institute of Technology, Hermann-von-Helmholtz-Platz 1, 76344 Eggenstein-Leopoldshafen, Germany; 2 Laboratory for Electron Microscopy, Karlsruhe Institute of Technology, Engesserstr. 7, 76131 Karlsruhe, Germany; 3 Department of Condensed Matter Physics, Charles University, Ke Karlovu 5, 121 16 Prague, Czech Republic; lukas.horak@matfyz.cuni.cz (L.H.);; 4 Institute for Applied Materials, Karlsruhe Institute of Technology, Hermann-von-Helmholtz-Platz 1, 76344 Eggenstein-Leopoldshafen, Germany; klaus.seemann@kit.edu (K.S.);; 5 Laboratory for Applications of Synchrotron Radiation, Karlsruhe Institute of Technology, Kaiserstr. 12, 76131 Karlsruhe, Germany

**Keywords:** h-YbFeO_3_, multiferroics, magnetic properties, PLD, TEM, XRD, microstructure, underlayer effect

## Abstract

The hexagonal ferrite h-YbFeO_3_ grown on YSZ(111) by pulsed laser deposition is foreseen as a promising single multiferroic candidate where ferroelectricity and antiferromagnetism coexist for future applications at low temperatures. We studied in detail the microstructure as well as the temperature dependence of the magnetic properties of the devices by comparing the heterostructures grown directly on YSZ(111) (i.e., YbPt_Th0nm) with h-YbFeO_3_ films deposited on substrates buffered with platinum Pt/YSZ(111) and in dependence on the Pt underlayer film thickness (i.e., YbPt_Th10nm, YbPt_Th40nm, YbPt_Th55nm, and YbPt_Th70nm). The goal was to deeply understand the importance of the crystal quality and morphology of the Pt underlayer for the h-YbFeO_3_ layer crystal quality, surface morphology, and the resulting physical properties. We demonstrate the relevance of homogeneity, continuity, and hillock formation of the Pt layer for the h-YbFeO_3_ microstructure in terms of crystal structure, mosaicity, grain boundaries, and defect distribution. The findings of transmission electron microscopy and X-ray diffraction reciprocal space mapping characterization enable us to conclude that an optimum film thickness for the Pt bottom electrode is *Th_Pt_* = 70 nm, which improves the crystal quality of h-YbFeO_3_ films grown on Pt-buffered YSZ(111) in comparison with h-YbFeO_3_ films grown on YSZ(111) (i.e., YbPt_Th0nm). The latter shows a disturbance in the crystal structure, in the up-and-down atomic arrangement of the ferroelectric domains, as well as in the Yb–Fe exchange interactions. Therefore, an enhancement in the remanent and in the total magnetization was obtained at low temperatures below 50 K for h-YbFeO_3_ films deposited on Pt-buffered substrates Pt/YSZ(111) when the Pt underlayer reached *Th_Pt_* = 70 nm.

## 1. Introduction

Epitaxial growth of rare earth hexaferrite (h-RFeO_3_, R = Y, Dy-Lu) thin films including h-YbFeO_3_ have recently attracted the attention of several researchers as it has been successfully stabilized in a metastable hexagonal structure (P63 cm) [1,2,3] by depositing it onto substrates with a hexagonal crystal structure like Al_2_O_3_ [4,5,6,7], YSZ(111) [6,7,8,9], MgO [5], and substrates buffered with platinum (Pt) [4,5,9], Fe_3_O_4_ [10], indium tin oxide (ITO) [8,9,11], SrTiO_3_ (STO), and others [7,12,13]. Jeong et al. [4] demonstrated the ferroelectricity of h-YbFeO_3_ films at room temperature (RT) for a film thickness of about 60 nm, where the films were grown by pulsed laser deposition (PLD) on sputtered Pt-buffered sapphire. In their case, the achieved ferroelectric polarization at RT was estimated as *Ps* = 15 µC/cm^2^ [4]. Furthermore, they also measured the magnetization hysteresis curves at different temperatures and they detected a magnetic ordering temperature at *T_N_* = 120 K [4].

There has been well-justified agreement derived from recent studies about the appearance of a new single multiferroic family where spontaneous electric and magnetic polarization simultaneously occur at low temperature in the hexagonal ferrites (h-RFeO_3_, R = Y, Dy-Lu) [2,4,8,9,11,13]. This family is becoming known by its ferroelectric and antiferromagnetic properties.

The goal of the magnetic properties characterization in relation to the structure was proved to be relevant for magneto-electric (ME) coupling, where the electrical modulation of the magnetic state in h-YbFeO_3_ single-phase multiferroic was very recently reported by Li et al. [13]. The recently increasing interest in the hexagonal ferrites reflects the potential to optimize h-YbFeO_3_-based devices for information storage and processing applications [14].

There have recently been several attempts to produce h-YbFeO_3_-based devices since the growth of a bottom electrode, such as Pt [4,5,9], ITO [8,9,11] or La_2/3_Sr_1/3_MnO_3_ (LSMO) [12,13], has become crucial for the characterization of ferroelectric and dielectric properties. Moreover, it is worthwhile to highlight the importance of tailoring the interfacial strain either by examining different substrates [7] or by introducing an underlayer, which could be simultaneously utilized as a bottom electrode. The modification of the misfit at the interface was proven by Zhang et al. [10] to affect the epitaxial quality and the magnetism of the grown h-YbFe_2_O_3_. Several investigations have been conducted on the microstructure of h-YbFeO_3_ grown on Al_2_O_3_ without an interlayer where misfit dislocations were revealed by means of high-resolution transmission electron microscopy (HRTEM) [5], and antiphase boundaries (API) were also detected in h-YbFeO_3_ grown on yttria-stabilized zirconia with [111] orientation, YSZ(111) [9]. Bauer et al. [3], using HRTEM combined with high-resolution X-ray diffraction (HRXRD), demonstrated the role of a PLD-grown Pt underlayer in the reduction in the mismatch between an h-LuFeO_3_ and Al_2_O_3_ substrate and decrease in the defect density, which led to improved crystal quality of h-LuFeO_3_. They also emphasized the necessity of an optimum thickness of the Pt underlayer to ensure the continuity and conductivity of the Pt bottom electrode [3]. In our opinion, there is a lack of deep understanding about the dependence of the h-YbFO_3_ microstructure on the growth parameters and particularly on the quality of the underlayer between the YbFeO_3_ film and the used substrate. Recently, Bauer et al. [15] demonstrated the relevance of the Pt underlayer quality for the crystal structure as well as the magnetic properties of hexaferrite BaFe_12_O_19_ films. They proved that PLD growth of an optimum platinum underlayer thickness significantly enhances the layer quality and the magnetic performance of the ferromagnetic layer.

In this work, we study in detail the film morphology and the microstructure of h- YbFeO_3_ grown directly on YSZ(111) and on Pt-buffered YSZ(111) with different Pt film thicknesses in order to explore the influence of the underlayer quality on the h-YbFeO_3_ crystal structure and on the resulting magnetic properties. For this purpose, we combined several characterization methods, including X-ray diffraction (XRD), atomic force microscopy (AFM), scanning and transmission electron microscopy (SEM/TEM), and superconducting quantum interference (SQUID) measurements. To the best of our knowledge, to date, the effect of the underlayer has not been considered in investigations related to h-YbFeO_3_ film.

## 2. Materials and Methods

### 2.1. PLD Growth of YbFeO_3_ on Pt-Buffered YSZ(111) Substrates

The Pt films were deposited on yttria-stabilized zirconia (YSZ) with [111] orientation in the PLD chamber in a vacuum environment after heating the substrate to *Tg* = 900 °C, using a different number of shots *N^Pt^sh* = 1647, 4100, 8235, 12,350, and 16,470, with a laser frequency of 5 Hz and an energy pulse of 60 mJ (i.e., fluence *F* = 16 J/cm^2^). Prior to the growth, the different substrates were cleaned by isopropanol and then annealed in the furnace for 2 h at a temperature of 1200 °C in order to achieve low roughness and good terrace morphology. The target was separated by 35 mm from the substrate. The samples were cooled slowly at 5 °C/min from *T^Pt^g* = 900 °C to room temperature (RT). The substrates used for the Pt growth were 8 mm × 8 mm in size. We divided the Pt/YSZ samples into two pieces for different subsequent treatments. In the case of one of the pieces with a size of 4 mm × 8 mm, ferroelectric (FE) YbFeO_3_ layers were grown on the different Pt bottom electrodes as mentioned above. For this purpose, the template (Pt/YSZ) was heated from RT to *T^YbFO^g* = 900 °C with a heating rate of 25 °C/min. The growth of the subsequent YbFeO_3_ layers was carried out in an oxygen atmosphere at a pressure of 400 mTorr and with a laser frequency of 1 Hz and number of shots *N^YbFO^sh* = 20,000. The deposition time was about 5 h. The Pt layer became encapsulated in the heterostructure system between YSZ and FE. In the manuscript, the corresponding samples with encapsulated Pt films of different thicknesses are named as follows: YbPt_Th0nm, YbPt_Th10nm, YbPt_Th40nm, YbPt_Th55nm, and YbPt_Th70nm. A summary of the PLD growth parameters is given in Table 1 section § 1 PLD growth conditions.

### 2.2. High-Resolution X-ray Diffraction Reciprocal Space Mapping

For the different Pt and YbFO reflections, 2D-reciprocal space maps (2D-HRXRD) were recorded using high-resolution diffraction at the NANO beamline, which is dedicated to the investigation of thin films and multilayer and nanostructured materials at the Karlsruhe institute of Technology KIT light source in Karlsruhe Germany. All the 2D-HRXRD data of the symmetric and asymmetric reflections were obtained by means of a Mythen linear detector positioned at the corresponding Bragg diffraction angles and by rocking the sample around the Bragg angle. All the X-ray measurements were performed at an energy *E* = 15 keV and a wavelength *λ* of 0.826 Å. Furthermore, azimuthal Phi(ϕ) scans were also measured by rotating the YbPt_Th0nm, YbPt_Th40nm, YbPt_Th55nm, and YbPt_Th70nm samples around the surface normal for the asymmetric reflection YbFO108.

### 2.3. X-ray Reflectivity and Profile Density

The specular X-ray reflectivity (XRR) was measured using a Rigaku Smartlab diffractometer (Rigaku, Tokyo, Japan). The data were recorded using Cu-K_α_ radiation with a wavelength *λ* = 1.5418 Å and a parallel beam with X-ray mirror. The measured data were evaluated by the well-known method for XRR analysis of multilayers with rough interfaces developed by Parratt [16]. The used model for fitting of the XRR curves of YbPt_Th0nm, YbPt_Th10nm, YbPt_Th40nm, YbPt_Th55nm, and YbPt_Th70nm is described as follows: a rough YSZ substrate, with a thin interlayer between the substrate and the first layer, and the first layer having a rough interface and rough top layer. The fit was carried out using a self-written script based on a least square fitting algorithm. All the fitting parameters are summarized in Table 1 section § 2. Film characterization.

### 2.4. Atomic Force Microscopy (AFM)

Ex situ AFM topography measurements were carried out in tapping mode with a Bruker Dimension ICON (Bruker, Karlsruhe, Germany) for the YSZ(111) substrate and the templates Pt_Th10nm, Pt_Th40nm, Pt_Th55nm, and Pt_Th70nm, as well as for the bilayer systems, such as YbPt_Th0nm, YbPt_Th10nm, YbPt_Th40nm, YbPt_Th55nm, and YbPt_Th70nm, after the subsequent growth of the YbFO layer. As sensors, we used OPUS AC160-NA cantilevers (NanoAndMore, Wetzlar, Germany) with force constants of 26 N/m and resonance frequencies of 300 kHz. The analysis of the AFM images was performed using the NanoScope v2 software package (Bruker, Karlsruhe, Germany). All the relevant derived topographical parameters are summarized in Table 1 section § 3. Film morphology.

### 2.5. Scanning Electron Microscopy (SEM) and Transmission Electron Microscopy (TEM)

The surface topography and the chemical composition of the samples YbPt_Th0nm, YbPt_Th10nm, YbPt_Th40nm, YbPt_Th55nm, and YbPt_Th70nm were characterized by SEM imaging combined with energy-dispersive X-ray spectroscopy (EDX) using an FEI DualBeam Helios G4 FX microscope (Thermo Fisher Scientific, Waltham, MA, USA). For YbPt_Th0nm only, the EDX maps were not recorded and are not presented here. For secondary electron (SE) imaging of the sample surface with 0 degrees of inclination, respectively, an Everhart–Thornley detector (ETD) was used. The microscope was operated at 10 kV accelerating voltage in the so-called field-free mode with a beam current of approximately 25 pA. In addition, to obtain element-specific information, backscattered electron (BSE) images were taken by means of a semiconductor (pn-diode) detector. Moreover, chemical analyses were performed by EDX at a primary electron energy of 20 keV and a beam current of 0.4 nA, applying a Bruker system of the type QUANTAX 400 with a silicon-drift detector (SDD) XFlash 6. For the samples YbPt_Th10nm, YbPt_Th40nm, YbPt_Th55nm, and YbPt_Th70nm, the two-dimensional distribution of the elements Pt, Zr, Yb, Fe, and O was imaged via mapping, where the acquisition time per map was about 5 min. By employing the ESPRIT 2.3 software, the raw-data X-ray maps were quantified using the thin-film approximation after Cliff–Lorimer [17] to obtain element-concentration maps.

For TEM inspection of the different samples YbPt_Th0nm, YbPt_Th40nm, YbPt_Th55nm, and YbPt_Th70nm, cross-sectional specimens were prepared by focused ion beam (FIB) milling using a FEI DualBeam Helios G4 FX microscope. Prior to FIB preparation, a thin gold layer was sputtered on the sample surface in order to reduce ion-beam damage of the heterostructures. Subsequently, standard FIB preparation of TEM lamellae was performed, where a Pt protection layer was deposited on top of the samples. Then, coarse FIB milling was carried out at a primary ion energy of 5 keV. The lamellae were attached to Cu lift-out grids and finally polished by a Ga+-ion beam with low energy of 1 keV to minimize Ga+ implantation and material amorphization. TEM investigations of all the above-mentioned samples were carried out on an aberration-corrected FEI Titan 80-300 microscope. This TEM has a thermally assisted field emission cathode (Schottky emitter) and was operated at 300 kV high voltage. For image recording, the microscope is equipped with a 4 k × 4 k CMOS camera of the F436 type (TVIPS). TEM bright-field imaging was performed to obtain information about general layer properties like, e.g., layer thickness and crystal structure (see Table 1 section § 2. Film characterization). Information about the crystal structure of the different materials in real space was obtained by conventional high-resolution TEM (HRTEM) and atomically resolved scanning TEM (STEM) using a high-angle annular dark-field (HAADF) detector.

### 2.6. Degree of Coverage

The values of the degree of coverage DoC (BSE) were obtained by processing the BSE images while the Yb-degree of coverage Yb_DoC (EDX) was determined from EDX maps of the element Yb. In detail, for this purpose, a Weka Trainable Segmentation plug-in of the ImageJ software ver. 3.2.33 [18] was utilized. Dark contrast groves were marked as substrate and regions with bright contrast as Yb. The software was iteratively trained by comparing the marked regions with the original image. The Weka Trainable Segmentation plug-in allows identifying the variable contrast regions. Image defects (such as contrast profile, charging contrast, etc.) could be eliminated by use of the iterative training option of the plug-in. The segmentation results were converted into binary images and the area fractions were measured by ImageJ and determined as the degree of coverage. All the estimated values are summarized in Table 1, section § 3. Film morphology.

### 2.7. Superconducting Quantum Interference Device (SQUID)

Out-of-plane (OOP) magnetization hysteresis loops were performed at temperatures *T* = 2 K, 10 K, 20 K, 30 K, 50 K, 100 K, 150 K, and 300 K using an MPMS3 SQUID magnetometer from the company Quantum Design for an applied field in the range of *B* = 0 to 6 Tesla. The measurements were selectively performed for the samples YbPt_Th0nm, YbPt_Th10nm, YbPt_Th55nm, and YbPt_Th70nm. Additionally, zero-field cooling (ZFC) and field cooling (FC) curves were also recorded, where the samples were cooled in the absence and the presence of the applied fields (*H* = 100 Oe, 2000 Oe, *H* is parallel to the c-axis), respectively, in the temperature range *T* = [2 − 300 K]. From the OOP magnetization loops, the saturation magnetization *Ms⊥(T)*, the remanent magnetization, *Mr⊥(T)*, and the OOP coercivity field *Hc⊥(T)* were derived and plotted versus temperature *T*.

## 3. Results

### 3.1. Dependence of the YbFeO_3_ Film Morphology on the Pt Underlayer Thickness

**Figure 1:**
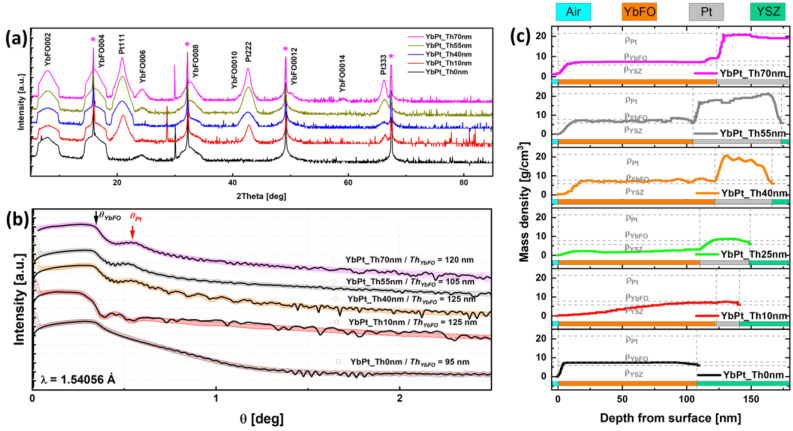
(**a**) XRD patterns of the samples YbPt_Th0nm, YbPt_Th10nm, YbPt_Th40nm, YbPt_Th55nm, and YbPt_Th70nm, where the magenta stars indicate the YSZ(111) peaks. The XRD curves are vertically shifted for better clarity. (**b**) XRR curves of the samples YbPt_Th0nm, YbPt_Th10nm, YbPt_Th40nm, YbPt_Th55nm, and YbPt_Th70nm, where *Q_YbFO_* and *Q_Pt_* are critical angles of the YbFO and Pt layers. XRR curves are vertically shifted for better visibility. Black solid lines represent the numerical simulations of the measured XRR curves. (**c**) Mass-density profiles along the film depth for the samples YbPt_Th0nm (**lowest panel**), YbPt_Th10nm, YbPt_Th40nm, YbPt_Th55nm, and YbPt_Th70nm (**upper panel**). The green, grey, orange, and turquoise bars correspond to the YSZ, Pt, YbFO, and air ranges for the different samples which are used for the determination of the layer thicknesses.

Figure 1a compares the diffraction patterns of YbFeO_3_ films grown directly on the YSZ(111) substrate, termed YbPt_Th0nm, and the YbFeO_3_ grown on Pt-buffered YSZ(111) with different platinum thicknesses *Th_Pt_*, termed YbPt_Th10nm, YbPt_Th40nm, YbPt_Th55nm, and YbPt_Th70nm. For enhanced clarity, the XRD patterns are vertically shifted.

In these patterns, we indicate the reflections of the substrates YSZ111, YSZ222, YSZ333, and YSZ444 by magenta stars, which were superposed for the different samples. We also identified the reflections corresponding to the Pt underlayer by Pt111, Pt222, and Pt333, where the intensity increases and the broadening decreases with *Th_Pt_*, indicating an enhancement in the degree of coverage of YSZ(111) and an improvement in the crystal quality of the Pt underlayer. It is obvious that the XRD pattern of YbPt_Th0nm, illustrated by a black solid line, does not contain any Pt reflection order. All the XRD patterns contain the different reflection orders YbFO002, YbFO004, YbFO006, YbFO008, YbFO0010, YbFO0012, and YbFO0014, confirming an epitaxial growth of hexagonal h-YbFeO_3_. However, the corresponding peak intensities and profiles are different, which reflects that the growth of the different h-YbFeO_3_ layers was influenced by the Pt underlayer thickness and quality. To better characterize the samples, XRR curves were simulated, and are depicted in Figure 1b, to determine the film thickness and the surface and interfaces roughness. In the XRR curves, we distinguish two critical angles, *Q_Pt_* = 0.55° and *Q_YbFO_* = 0.34°, for the samples YbPt_Th10nm, YbPt_Th40nm, YbPt_Th55nm, and YbPt_Th70nm, and one critical angle, *Q_YbFO_* = 0.34°, for the sample YbPt_Th0nm. From the fitting of the XRR curves, we derived the film thickness of the Pt and YbFeO_3_ layers in the heterostructure systems, where Pt is the encapsulated state between the YbFeO_3_ and YSZ(111) (see section § 2. Film characterization in Table 1). The mass density profiles *ρ_XRR_* derived from the XRR simulation are illustrated in Figure 1c, where the bottom and upper panels correspond to YbPt_Th0nm and YbPt_Th70nm, respectively. We differentiate the different layers composing the heterostructure systems by using a green legend for the YSZ(111) substrate, grey for the Pt film, and orange for the YbFO layer.

It should be emphasized that all the YbFeO_3_ films on Pt-buffered YSZ(111) with different platinum thicknesses *Th_Pt_* = 10, 40, 55, and 70 nm were grown under the same conditions, such as the growth temperature *T^YbFO^g* = 900 °C, oxygen pressure *P_O2_* of 400 mTorr, and number of shots *Nsh* = 20000. From the mass density profiles *ρ_XRR_*, it is possible to determine the thickness of the different layers in the samples (see Table 1, section § 2. Film characterization).

For sample YbPt_Th0nm, we found an YbFeO_3_ film thickness *Th_YbFO_* = 95 ± 5 nm, while for YbPt_Th40nm *Th_YbFO_* it was 125 ± 5 nm, *Th_YbFO_* = 105 ± 5 nm for YbPt_Th55nm, and, finally, *Th_YbFO_* = 120 ± 5 nm for YbPt_Th70nm. These results indicate that the Pt underlayer thickness *Th_Pt_* slightly influences the growth kinetics of the subsequent YbFO layer. In fact, the samples YbPt_Th0nm and YbPt_Th55nm had comparable thicknesses *Th_YbFO_* in the uncertainty range. If we compare the corresponding XRD diffraction patterns (i.e., black solid line for YbPt_Th0nm and olive-green solid line for YbPt_Th55nm), we deduce a reduction in the broadening of the diffraction peak YbFO002, which indicates an enhancement in the YbFO crystal quality. This issue needs to be further investigated in more detail.

**Figure 2:**
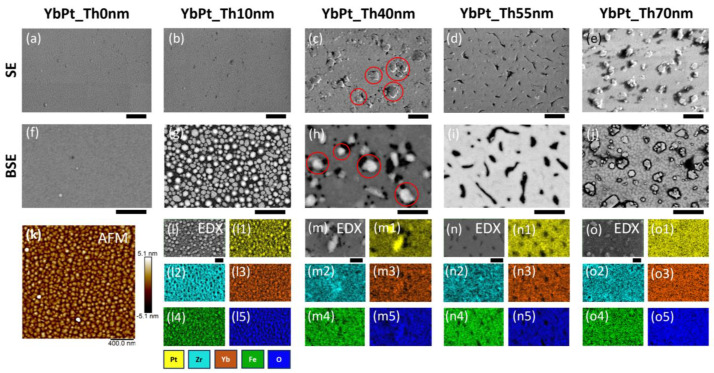
(**a**–**e**) and (**f**–**j**) SEM and BSE images of the samples YbPt_Th0nm, YbPt_Th10nm, YbPt_Th40nm, YbPt_Th55nm, and YbPt_Th70nm, with a scale bar of 1 µm. (**k**) AFM image of the YbPt_Th0 nm film, with a scale bar of 400 nm. (**l**,**l1**–**l5**), (**m**,**m1**–**m5**), (**n**,**n1**–**n5**) and (**o**,**o1**–**o5**) are the EDX maps of the elements Pt, Zr, Yb, Fe, and O for the samples YbPt_Th10nm, YbPt_Th40nm, YbPt_Th55nm, and YbPt_Th70nm, respectively, with a scale bar of 500 nm. Red circles refer to the hillocks on the surface.

Regarding the mass density of YbFO, for the sample YbPt_Th0nm, the value *ρ_YbFO_* = 7.82 g/cm^3^ corresponds to a continuous YbFeO_3_layer, while for the sample YbPt_Th10nm, the mass density *ρ_YbFO_* is lower than *ρ_YbFeO3_* = 7.82 g/cm^3^. This is probably due to the lower degree of coverage of Pt for *Th_Pt_* = 10 nm, which influences the YbFO morphology. In the following, this issue will be discussed in more detail. As the *Th_Pt_* increases beyond 40 nm, the degree of coverage increases and the Pt-buffered YSZ(111) becomes more homogeneous, and able to withstand the dewetting phenomenon which occurs during the subsequent YbFO growth. Nergis et al. [19] demonstrated the structural and morphological modification of the platinum underlayer in the as-grown and encapsulated states. They revealed the occurrence of competitive phenomena in the Pt film grown by PLD on YSZ(111), such as dewetting, hillock formation, and filling of the voids separating the channels in the Pt network by Pt migration due to high diffusion energy during the subsequent growth of the capping layer at *Tg* = 900 °C. It is worthwhile to compare the morphology of the YbFO films as a function of the Pt underlayer thickness, as illustrated in Figure 2. Here, the upper panel shows SE images of the samples YbPt_Th10nm, YbPt_Th40nm, YbPt_Th55nm, and YbPt_Th70nm. The corresponding BSE images are given in the panels of the second row, and, finally, EDX maps are shown in the third, fourth, and fifth rows of Figure 2.

**Figure 3:**
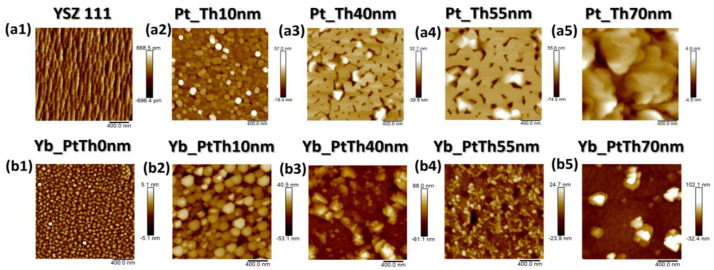
(**a1**–**a5**) AFM images with a size of 2 µm × 2 µm and a scale bar of 400 nm for the samples YSZ111, Pt_Th10nm, Pt_Th40nm, Pt_Th55nm and Pt_Th70nm, respectively. (**b1**–**b5**) are the corresponding AFM images of the samples after the subsequent growth of YbFO layers with 20,000 shots for the samples Yb_PtTh0nm, Yb_PtTh10nm, Yb_PtTh40nm, Yb_PtTh55nm, and Yb_PtTh70nm, respectively.

It should be noted that, depending on the Pt underlayer thickness, there is a visible modification of the morphology and element distribution of the different PLD-grown YbFO films. In the upper panel of Figure 3, AFM images of the Pt-buffered YSZ(111) in the as-grown state are shown, while the bottom panel presents the resulting morphology of the corresponding YbFO films grown on these templates. The goal is to point out the modifications of the Pt underlayer occurring during the subsequent growth, which has been highlighted and discussed in detail by Nergis et al. [19]. An important issue is the hillock formation, which was pronounced in the case of *Th_Pt_* = 40 nm and remained even after the deposition of the YbFO layer, as indicated by the red circles drawn on the regions with bright contrast in Figure 2c,h. The EDX maps of the Pt, Yb, and Fe given in Figure 2(m1,m3,m4) confirm that the Pt hillocks were also covered by the YbFO layer, but there were some voids which were free of Pt and Yb, represented by black regions in Figure 2(m1,m3).

In a complementary way, Figure 3(a3,b3) prove that the hillocks, which were formed in the Pt-buffered YSZ(111) in the as-grown state, persist during the YbFO growth and affect the crystal quality of the YbFO layer by creating constraints in the YbFO mosaic blocks. In contrast, for *Th_Pt_* = 55 nm, the hillocks formed in the as-grown state of the Pt-buffered YSZ(111) migrate toward the holes to generate a homogeneous Pt underlayer as demonstrated by Figure 3(a4,b4). In consequence, we obtain an YbFO film network which contains Pt- and Yb-free holes (see Figure 2d,i,(n1,n3)). Even though, YbPt_Th0nm and YbPt_Th55nm have a comparable YbFO film thickness *Th_YbFO_* (TEM) ≅ 100 ± 5 nm, the film morphology is islands for YbPt_Th0nm (Figure 3(b1)) and a 2D-continuous network + holes for YbPt_Th55nm (Figure 3(b4)). The YbFO film morphology shown in Figure 2j and Figure 3(b5) indicate two levels of growth, consisting first of a continuous and homogeneous YbFO film, followed by agglomerates of YbFO when *Th_YbFO_* exceeds 100 nm. In order to avoid the overgrowth and to preserve the continuity and homogeneity of the YbFO layer, it is necessary to reduce the number of shots *Nsh* to be lower than 20,000 for *Th_Pt_* = 70 nm (cf. Figure 3(b5)). From the findings of complementary methods using microscopy methods such as SEM, BSE, and AFM and XRR measurements, we demonstrate that the Pt underlayer morphology evolves from 3D-island growth to a 2D network containing holes in a homogenous and continuous film when *Th_Pt_* exceeds 55 nm thickness. This consequently affected the surface morphology and the homogeneity of the YbFO film and Yb element chemical distribution as well as the mass density of the YbFO obtained film, which reached the stoichiometry of the YbFeO_3_ target (*ρ_YbFeO3_* = 7.82 g/cm^3^) when the Pt underlayer was grown with its optimum thickness, as illustrated in Figure 1c and Figure 2(o3).

### 3.2. Effect of the Pt Underlayer on the Crystal Structure and Mosaicity

**Figure 4:**
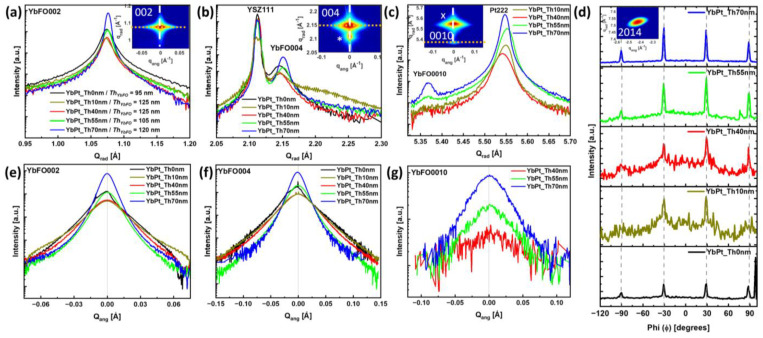
(**a**–**c**,**e**–**g**) Comparison of the radial and the angular diffraction profiles corresponding to the samples Yb_PtTh0nm, Yb_PtTh10nm, Yb_PtTh40nm, Yb_PtTh55nm, and Yb_PtTh70nm for the reflections YbFO002, YbFO004, and YbFO0010, respectively. (**d**) Layout of the azimuthal scans I (ϕ) of the asymmetric reflection YbFO2014 for Yb_PtTh0nm, Yb_PtTh10nm, Yb_PtTh40nm, Yb_PtTh55nm and Yb_PtTh70nm, ordered from bottom to top panel, respectively.

**Figure 5:**
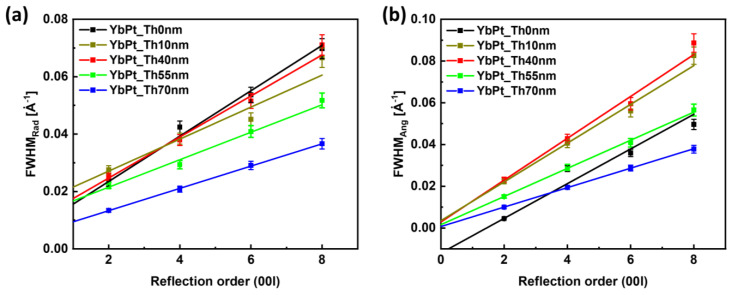
Variation in the *FWHM_rad_* (**a**) and *FWHM_ang_* (**b**) with the reflection order 00l for the samples Yb_PtTh0nm, Yb_PtTh10nm, Yb_PtTh40nm, Yb_PtTh55nm, and Yb_PtTh70nm for the determination of the mosaicity parameters. WH approach for YbPt_Th0nm sample is non-applicable (NA) along the radial direction, since the y-intercept falls below 0. The regression line is used only for illustration of the linearity.

Figure 4a–c compare the radial diffraction profiles along the crystal truncation rod of selected reflections, such as YbFO002, YbFO004, and YbFO0010, respectively, for the different samples YbPt_Th0nm, YbPt_Th40nm, YbPt_Th55nm, and YbPt_Th70nm. The radial diffraction profiles corresponding to YbFO002, YbFO004, YbFO006, and YbFO008 were fitted with Pseudo-Voigt functions using the non-linear square algorithm. We derived the peak positions and the full width-half maximum *FWHM_rad_*, which is plotted against the reflection order in Figure 5a. Figure 4b includes the diffraction peaks YSZ111 of the substrate, which were superposed for the different samples and used as a reference to compare the intensities and the broadening of the diffraction peaks of the YbFO crystal. From Figure 4a–c, we deduce that YbPt_Th40nm and YbPt_Th70nm have the lowest and the highest peak intensities, respectively. Additionally, the *FWHM_rad_* seems to be larger in the case of sample YbPt_Th0nm. Furthermore, the *FWHM_rad_* decreases as the thickness of the Pt underlayer *Th_Pt_* increases, which reflects an improvement in the layer quality, as demonstrated by Figure 5a.

It is worthwhile to point out that the peak intensity of Pt222 in Figure 4c increases with the Pt underlayer thickness *Th_Pt_*, which confirms the improvement in the degree of coverage, as demonstrated by Figure 3. Figure 4d, presenting the azimuthal scan of the asymmetric reflection YbFO2014, shows that the YbFO layers are epitaxially grown with six-fold hexagonal symmetry for all the investigated samples. However, the peak intensities become more pronounced as the Pt underlayer thickness *Th_Pt_* increases, which clearly indicates the improvement in the YbFO crystal quality induced by the Pt underlayer.

Consequently, YbFO is expected to exhibit ferroelectricity along the growth c-axis of the noncentrosymmetric hexagonal cell, as demonstrated by Jeong et al. [4] in the case of YbFO films with a thickness of 60 nm grown on Pt(111)/sapphire(0001), where Pt(111) was rather sputtered. No information was given about the thickness of the Pt bottom electrode. The low peak intensities recorded in the azimuthal scan of Figure 4d in the case of YbPt_Th0nm in comparison with YbPt_Th70nm point to a probable weak ferroelectricity and disturbance in the ferroelectric domains in the YbFO layer of sample YbPt_Th0nm.

From the coordinates of the symmetric reflections (i.e., YbFO002, YbFO004, YbFO006, and YbFO008) and the asymmetric reflections (YbFO2012, YbFO2013, and YbFO2014), we determined the in-plane *a_YbFO_* and out-of-plane *c_YbFO_* lattice parameters from the hexagonal structure formulas. Moreover, the misfit *f_YbFO/YSZ_* between the substrate YSZ(111) and the film was calculated for the sample YbPt_Th0nm using the 3 × d(11-2)YSZ formula of the substrate to the *a_YbFO_* parameter of the layer. This is because of the 3-to-1 coincidence in the lattice site between the substrate YSZ(111) and the film YbFO.

(1) $$f_{YbFO/YSZ}\left[\%\right]=\frac{a_{YbFO}-{3*d(11\bar{2})}_{YSZ}}{3{\;*\;d(11\bar{2})}_{YSZ}}*100$$

In fact, the in-plane epitaxial atomic orientation recorded during the growth of h-YbFeO_3_ on YSZ(111) involves the alignment of the (100) planes of YbFO parallel to the (11-2) ones of YSZ, which gives a misfit value of *f_YbFO/YSZ_* = −5.32% (see Table 2, section § 4. Crystal structure). A comparable value was reported by Xu et al. [2] for h-LuFeO_3_ grown on YSZ(111), where the oxygen network of h-LuFeO_3_ and YSZ(111) matched at the interface with a misfit of *f_LuFeO3/YSZ_* = −5.6%, creating a strong interfacial bonding. It should be emphasized that h-LuFeO_3_ and h-YbFeO_3_ are isostructural and have similar lattice parameters [10].

On the other side, the misfit *f_YbFO/Pt_* between the Pt underlayer and the YbFO film was calculated for the samples YbPt_Th10nm, YbPt_Th40nm, YbPt_Th55nm, and YbPt_Th70nm using the 4 × *d(11-2)Pt* formula of the Pt underlayer to the *a_YbFO_* parameter of the YbFO layer. This is because of the 4-to-1 coincidence in the lattice site between the Pt(111) substrate and the YbFO film.

(2) $$f_{YbFO/Pt}\left[\%\right]=\frac{a_{YbFO}-{4*d(11\bar{2})}_{Pt}}{4{\;*\;d(11\bar{2})}_{Pt}}*100$$

We found that the misfit of *f_YbFO/Pt_* gradually decreases in absolute value from *f_YbFO/Pt_* = −8.46% to −6.83% and becomes closer to the misfit value of *f_YbFO/YSZ_* = −5.32% (see Table 2, section § 4. Crystal structure) as *Th_Pt_* increases, which demonstrates the enhancement of the matching between the atomic networks of YbFO and Pt(111) at the interface when the Pt underlayer becomes more continuous and homogeneous. A misfit value of *f_LuFeO3/Pt_* = −7.5% was also reported by Xu et al. [2] without any information provided about the platinum film thickness.

The in-plane *ε^//^_YbFO_* and out-of-plane residual strain *ε^⊥^_YbFO_* (see Table 2, section § 4. Crystal structure) are calculated using the following formulas:

(3) $$\;\varepsilon_{YbFO}^{//}\;\left[\%\right]=\frac{a_{YbFO-a_{YbFO}^{FS}}}{a_{YbFO}^{FS}}*100\;$$

(4) $$\mathrm{and}\;\varepsilon_{YbFO}^{⊥}\;\left[\%\right]=\frac{c_{YbFO-c_{YbFO}^{FS}}}{a_{YbFO}^{FS}}*100$$

*a_YbFO_* and *c_YbFO_* are the in-plane and out-of-plane lattice parameters of the YbFO layer, respectively. $a_{YbFO\;}^{FS}=5.9652\;Å$ and $c_{YbFO\;}^{FS}=11.7020\;Å$ (ICSD 183152, space group P63cm) correspond to the lattice parameters of the YbFO bulk in the free-standing state (FS). All the derived structural parameters are listed in Table 2, section § 4. Crystal structure.

Figure 4e–g present the angular diffraction profiles of the reflections YbFO002, YbFO004 and YbFO0010, respectively, for the different samples YbPt_Th0nm, YbPt_Th40nm, YbPt_Th55nm, and YbPt_Th70nm. In a similar way, the profiles were fitted and the angular *FWHM_ang_* was determined and plotted as a function of the reflection order, as shown in Figure 5b. We deduce that *FWHM_ang_* was the largest for sample YbPt_Th0nm in comparison to YbPt_Th70nm. This permits us to draw conclusions about the enhancement of crystal quality of the epitaxial YbFO film as *Th_Pt_* increases and reaches the optimum *Th_Pt_* = 70 nm. We applied the Williamson–Hall (WH) approach [19,20,21] to the plots of Figure 5a,b, where we assumed that the YbFO film is composed of mosaic blocks with a lateral size *L^H^_YbFO_* and vertical size *L^V^_YbFO_*, which were misaligned relative to the c-axis with a degree of misorientation *α_YbFO_* and vertically strained due to defect and grain boundary formation. From the intercept and the slope of the different *FWHM_rad_* plots vs. the reflection order in Figure 5a, we derived the mean vertical size *L^V^_YbFO_* and mean values of the vertical strain distribution <*β^⊥^_YbFO_*> of the YbFO mosaic crystal blocks, as summarized in section § 5. Mosaicity of Table 2. This latter shows that *L^V^_YbFO_* was found to be 81.8 nm ± 5 nm and 100.35 ± 5 nm for the cases of YbPt_Th0nm and YbPt_Th70nm, respectively, which was the same order as the film thickness *Th_YbFO_* (TEM) = 95 ± 5 nm. This leads to the conclusion that the YbFO crystal extends along the whole film thickness. Oppositely, for other samples YbPt_Th40nm and YbPt_Th55nm, the *L^V^_YbFO_* is lower than the YbFO film thickness *Th_YbFO_* (TEM), as can be seen by comparing sections §2. Film characterization of Table 1 and §5. Mosaicity of Table 2. The WH approach applied to the radial diffraction profiles was used to determine the contribution of the vertical size *L^V^_YbFO_* and the vertical strain contribution <*β^⊥^_YbFO_*> to the radial broadening *FWHM_rad_*, which was pronounced in the case of YbPt_Th0nm without a Pt underlayer, as shown in Figure 4a–c.

Furthermore, <*β^⊥^_YbFO_*> is 3.95 × 10^−3^ for YbPt_Th0nm, while for YbPt_Th70nm <*β^⊥^_YbFO_*> = 1.82 × 10^−3^, which argues for the presence of a high mean value for the vertical strain distribution in the absence of a Pt underlayer and improvement in the YbFO layer quality by the deposition of an underlayer with an optimum *Th_Pt_*. The vertical strain not only depends on the Pt thickness *Th_Pt_*, but also on the underlayer quality, where the hillock formation for YbPt_Th40nm demonstrated by Figure 2j also increased the vertical strain to <*β^⊥^_YbFO_*> = 3.58 × 10^−3^. Similarly, from the intercept and the slope of the different FWHM_ang_ plots vs. the reflection order in Figure 5b, we determined the degree of misorientation α_YbFO_ and the mean value of lateral size *L^H^_YbFO_* of the YbFO crystal mosaic blocks for the different samples and we list them in section § 5. Mosaicity in Table 2. The *α_YbFO_* can be considered as a measure of defect density in the YbFO layer. The low values of *α_YbFO_* < 0.5 deg for YbPt_Th70nm indicate good quality of the YbFO layer, while the high values of *α_YbFO_* > 0.5 deg for YbPt_Th0nm, YbPt_Th40nm, and YbPt_Th55nm reflect the existence of a high defect density and the presence of constraints in the film, such as hillocks or grain boundaries, which will be discussed below on the basis of the TEM results. Furthermore, the increase in the *L^H^_YbFO_* of the YbFO crystal mosaic blocks reflects a decrease in the number of grain boundaries. We found that *L^H^_YbFO_* = 571.2 ± 5 nm for YbPt_Th70nm and *L^H^_YbFO_* = 218.2 ± 5 nm for YbPt_Th40nm. This latter gives a hint of the existence of a high number of grain boundaries in YbFO for the sample YbPt_Th40nm (Table 2, section § 5. Mosaicity). This also applies in the case of YbPt_Th10nm with *L^H^_YbFO_* = 179 ± 5 nm.

In conclusion, the crystal YbFO quality is significantly enhanced when *Th_Pt_* = 70 nm, as is demonstrated by the remarkable decrease in *FWHM_ang_*, as well as by the reduction in the number of grain boundaries, which are interrelated with the expansion of the lateral size of the YbFO mosaic blcoks *L^H^_YbFO_* and the decrease in the degree of misorientation. These structural characteristics were not achieved when the *Th_Pt_* was below 55 nm.

### 3.3. Influence of the Pt Underlayer on the YbFeO_3_ Microstructure

**Figure 6:**
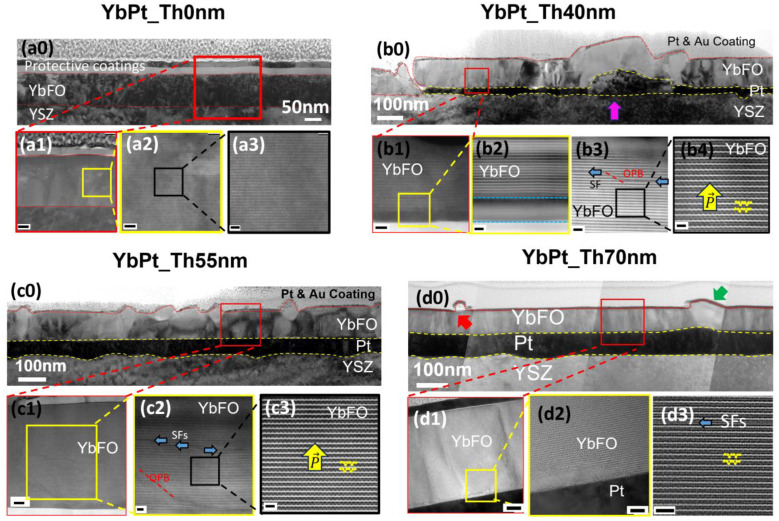
(**a0**–**a3**) TEM images for YbPt_Th0nm, where (**a0**) image is the overview TEM with the lowest magnification and scale bar of 50 nm, (**a1**) image corresponds to the red rectangular region drawn in (**a0**), (**a2**) image corresponds to the yellow square drawn in (**a1**), and (**a3**) is the high-magnification images of the black rectangular region shown in (**a2**). (**b0**–**b4**) TEM images for YbPt_Th40nm, where (**b0**) image is the overview TEM with the lowest magnification and scale bar of 100 nm, (**b1**) image corresponds to the red rectangle region drawn in (**b0**), **(b2**) image corresponds to the yellow square drawn in (**b1**), and (**b4**) is the high-magnification images of the black rectangular region shown in (**b3**). (**c0**–**c3**) TEM images for YbPt_Th55nm, where (**c0**) image is the overview TEM with the lowest magnification and scale bar of 100 nm, (**c1**) image corresponds to the red rectangular region drawn in (**c0**), (**c2**) image corresponds to the yellow square drawn in (**c1**), and (**c3**) is the high-magnification images of the black rectangular region shown in (**c2**). (**d0**–**d3**) TEM images for YbPt_Th55nm, where (**d0**) image is the overview TEM with the lowest magnification and scale bar of 100 nm, (**d1**) image corresponds to the red rectangular region drawn in (**d0**), (**d2**) image corresponds to the yellow square drawn in (**d1**), and (**d3**) is the high-magnification images of the black rectangular region shown in (**d2**). Scale bars of (**a1**), (**a2**) and (**a3**) are 25 nm, 5 nm, and 2 nm, respectively. Scale bars of (**b1**), (**b2**), (**b3**), and (**b4**) are 5 nm, 2 nm, 2 nm, and 1 nm, respectively. Scale bars of (**c1**), (**c2**), and (**c3**) are 10 nm, 5 nm, and 1 nm, respectively. Scale bars of (**d1**), (**d2**), and (**d3**) are 10 nm, 5 nm, and 2 nm, respectively.

Figure 6 presents cross-sectional TEM images of the samples YbPt_Th0nm, YbPt_Th40nm, YbPt_Th55nm, and YbPt_Th70nm in order to evaluate the quality of the Pt underlayer and the post-grown YbFO films, and to show how the underlying Pt-layer quality influenced the microstructure of the subsequent YbFO layers. This comparison will be discussed with respect to YbPt_Th0nm, which does not contain any Pt underlayer. From the TEM images, we determined the layer thickness of the Pt *Th_Pt_* (TEM) and YbFO layers *Th_YbFO_* (TEM) (see Table 1, section § 2. Film characterization).

We deduce that *Th_YbFO_* (TEM) = 95 ± 5 nm of the YbFO layers is of the same order as for YbPt_Th0nm and YbPt_Th70nm since the Pt underlayer in Figure 6(d0) appears as a continuous layer with less thickness fluctuations. The mosaicity study discussed in the previous chapter confirmed that the YbFO quality in YbPt_Th0nm is lower than in YbPt_Th70nm, which led to a high degree of misorientation *α_YbFO_* = 0.93 ± 0.05 deg and a large mean value of the vertical strain distribution *<β^⊥^_YbFO_>* = 3.95 × 10^−3^ ± 0.5 × 10^−4^. These values were almost halved by the deposition of a continuous Pt underlayer in the case of YbPt_Th70nm (see section § 5. Mosaicity of Table 2). This indicates that the YbFO layer of sample YbPt_Th0nm has a higher defect density than that of sample YbPt_Th70nm. Consequently, the TEM images of the YbPt_Th0nm and YbPt_Th70nm samples in Figure 6(a0,d0) exhibit different image contrasts. Furthermore, although samples YbPt_Th0nm and YbPt_Th70nm have the same YbFO layer thickness *Th_YbFO_* (TEM) = 95 ± 5 nm, the diffraction intensities of YbFO002, YbFO004, and YbFO008 shown in Figure 1a and Figure 4a–c are intense in the case of YbPt_Th70nm and weaker in the case of YbPt_Th0nm. The HRTEM images of Figure 6(a1,a2,d1,d2), which correspond to the YbPt_Th0nm and YbPt_Th70nm samples, respectively, hint at a disturbance of the YbFO atomic arrangement for sample YbPt_Th0nm, which appears as a blurring of the image-contrast effect in the TEM images of Figure 6(a2,a3). This can induce a loss of the coherent diffracted intensities that are usually produced by the well-ordered YbFO crystal lattice planes.

Regarding the intermediate Pt underlayer thicknesses, *Th_Pt_* = 10, 40, and 55 nm, TEM studies were conducted on samples YbPt_Th40nm and YbPt_Th55nm, as shown in Figure 6(b0)–(b4) and Figure 6(c1–c3), respectively. The morphological study performed by AFM, SEM, and BSE imaging discussed in chapter § 3.1 demonstrates different morphologies for the YbPt_Th40nm and YbPt_Th55nm samples, mainly due to hillock formation, which was more predominant in the case of YbPt_Th40nm (cf. Figure 2h,i). The cross-sectional TEM images in Figure 6(b0) confirm strong fluctuation in the Pt underlayer film thickness where *Th_Pt_* (TEM) varies between 18 and 96 ± 5 nm (section § 2. Film characterization in Table 1) due to the presence of hillocks, indicated by a magenta arrow in Figure 6(b0). In contrast, the TEM images of Figure 6(c0) of YbPt_Th55nm do not reveal the presence of hillocks in the Pt underlayer and indicate the deposition of a more or less continuous Pt layer with less pronounced fluctuation in the layer thickness (i.e., *Th_Pt_* (TEM) = 56.5 ± 5 nm in Table 1 and Table 2) compared to YbPt_Th40nm. Furthermore, the presence of hillocks in YbPt_Th40nm has strongly influenced the microstructure of the YbFO layer, which contains some discontinuity regions and lateral phase boundaries, deducible from the TEM contrast in Figure 6(b0). The TEM image of Figure 6(b1) corresponds to the red rectangle drawn in the interface region of the YbFO/Pt of Figure 6(b0). High-resolution TEM images in Figure 6(b2,b3) demonstrate the formation of stacking-fault (SF) defects and out-of-phase boundaries (OPB) in addition to a smeared interface with the Pt underlayer for sample YbPt_Th40nm. The local findings on the microstructure are in accordance with a high degree of misorientation *α_YbFO_* = 1.07 ± 0.05 and the mean vertical strain distribution *<β^⊥^_YbFO_>* = 3.58 × 10^−3^ ± 0.5 × 10^−4^. The increase in the Pt underlayer thickness from *Th_Pt_* = 40 to 55 nm significantly improved the continuity of the film and its morphology as well as the quality of the Pt crystallinity by preventing the persistence of hillocks within the Pt layer. This is in return, reduced the characteristic mosaicity values of the misorientation and vertical strain, as illustrated in Table 2 section § 5. Mosaicity.

Figure 6(b4,c3) correspond to two selected regions in the TEM images of Figure 6(b3,c2), which are marked by black rectangular areas outside the SF and OPB defect regions. An up-and-down atomic arrangement of the ferroelectric domains was revealed for the samples YbPt_Th40nm and YbPt_Th55nm in the high-resolution HAADF STEM images with nm bar scales. This ordering was completely disturbed for the sample YbPt_Th0nm, as can be observed in Figure 6(a3).

The results derived from X-ray diffraction and the microscopy methods are very complementary and in accordance with each other. They emphasize the importance of the crystal quality of the Pt underlayer, which could only be achieved when the Pt thickness was beyond *Th_Pt_* = 55 nm. The optimization of the Pt underlayer in the case of YbPt_Th70nm enabled us to demonstrate the improvement in the YbFO crystal quality in terms of a reduction in the defect density and in the disturbance of the atomic arrangement, as confirmed by the high-resolution TEM and STEM imaging.

The study of the YbFO microstructure by means of HRTEM enables deep understanding of the relevance of the Pt underlayer microstructure, which suffers from discontinuity, and the problem of hillocks formation when *Th_Pt_* does not achieve an optimum thickness. This results in disturbance in the YbFO crystal quality as well as to the formation of defects, which inhibit the ferroelectric performance. Even though outside the defect regions, we could clearly visualize the presence of ferroelectric domains.

### 3.4. Modification of the Antiferromagnetic Properties with the Pt Underlayer Quality

**Figure 7:**
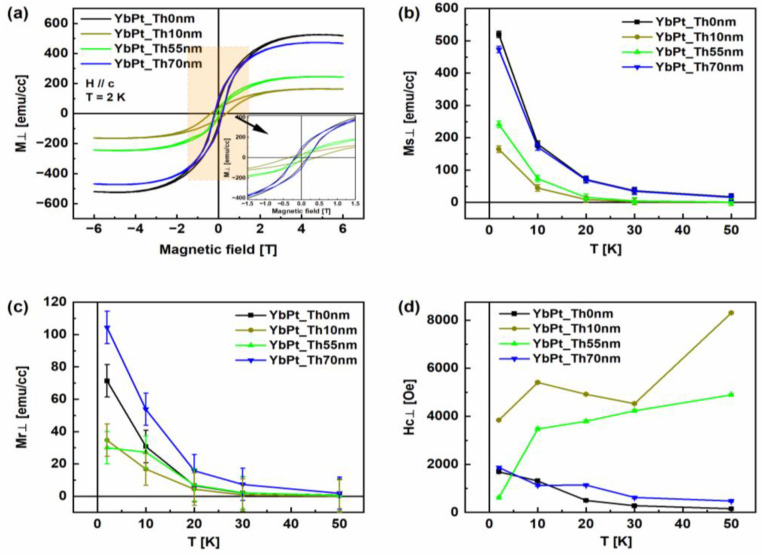
(**a**) Out-of-plane (OOP) magnetization hysteresis loops measured at 2 K for an applied field up to 6 tesla for the samples YbPt_Th0nm, YbPt_Th10nm, YbPt_Th55nm, and YbPt_Th70nm. Dependence of the magnetization at saturation *Ms⊥* (**b**), remanent magnetization *Mr⊥* (**c**) and perpendicular coercivity *Hc⊥* (**d**) on the temperature *T* for the samples YbPt_Th0nm, YbPt_Th10nm, YbPt_Th55nm, and YbPt_Th70nm.

Magnetization measurements using the SQUID device were selectively performed for the samples YbPt_Th0nm, YbPt_Th10nm, YbPt_Th55nm, and YbPt_Th70nm. The OOP magnetization hysteresis curves were measured for each sample at different temperatures of *T* = 2 K, 10 K, 20 K, 30 K, 50 K, 100 K, 150 K, and 300 K. From the OOP curves, the magnetization at saturation *Ms⊥*, remanent magnetization *Mr⊥*, and OOP coercivity *Hc⊥* were derived and plotted versus temperature for the different samples, as shown in Figure 7b–d, respectively. Figure 7b shows the non-linear increase in *Ms⊥* with *Th_Pt_* at each temperature. For illustration, the OOP magnetic hysteresis loops are shown for the different samples for *T* = 2 K in Figure 7a, where the *Ms⊥* of the samples YbPt_Th0nm and YbPt_Th70nm are slightly different and are lower for YbPt_Th10nm and YbPt_Th55nm due to the imperfections of the YbFO layer as revealed by our structure studies. The inset in Figure 7a presents a magnification of the hysteresis loops to clearly visualize the difference between the remanent moments *Mr⊥* which vary between the different samples. It should be noted that the hysteresis effect was not detected for all the measured samples above *T* = 100 K. This result is in accordance with previous investigations carried out on the magnetic properties of h-YbFeO_3_ [2,4,6,9]. It should be emphasized that the total magnetic moments *Ms⊥* at low temperatures have contributions from the Yb^3+^ and Fe^3+^ sites and their interactions (Fe–Fe, Fe–Yb, and Yb–Yb) where the Fe^3+^ spins order at first through the Fe–Fe interaction, then the spins of paramagnetic Yb^3+^ sites are polarized by the exchange field originated from Fe^3+^ and, therefore, from the Yb–Fe interaction, and, finally, the long-range order of Yb^3+^ is established through the Yb–Yb interaction [2,9,22]. Based on the formalism of Cao et al. [22], the total magnetization can be expressed as follows:

(5) $$M=M_{Fe}*\left(1+\frac{\chi_{Yb}*\Gamma_{YbFe}}{\mu_{Yb}}\right)+\chi_{Yb}*H$$

where Γ*_YbFe_* is the molecular field of the Yb–Fe interaction, H is the external applied field, χ*_Yb_* is the susceptibility, while μ*_Yb_* is the magnetic moment of Yb.

Figure 7b,c show an increase in the magnitude of *Ms⊥* and *Mr⊥* as the temperature decreases independently of the *Th_Pt_.* This rise became more rapid when the temperature was lower than 20 K, which indicates the strong contribution due to the spin ordering of Yb^3+^ induced by the molecular field of Fe^3+^. Similar behavior was previously reported in the case of h-YbFeO_3_ grown on YSZ(111) without an underlayer [9]. In previous studies, the temperature dependences of *Ms⊥* and *Mr⊥* were not discussed in relation to the effect of the buffer layer as we seek to do. In Figure 7b, the temperature dependence of the magnetization reveals a significant influence of *Th_Pt_* at low temperatures of *T* = 2 K, 10 K. Moreover, the behavior of *Ms⊥* in relation to temperature was found to be comparable for the samples YbPt_Th0nm and YbPt_Th70nm. However, the remanent magnetization *Mr⊥* (*H* = 0) was remarkably improved by enhancing the quality of the YbFO layer, which underlies Pt in the case of YbPt_Th70nm, as shown in Figure 7c. From Equation (5) at *H* = 0, the total moment *Mr⊥* depends on Γ*_YbFe_*, the molecular field of the Yb–Fe interaction, as well as on *M_Fe_*. The enhancement of the *Mr⊥* for YbPt_Th70nm is interrelated with the strong Yb–Fe spins interaction, which is influenced by the quality of the atomic arrangement. Figure 6(a0–a3) reveal a disturbance in the atomic arrangement, which could degrade the strength of the Yb–Fe interactions and explain the reduction in *Mr⊥* in the case of YbPt_Th0nm. On the other hand, crystal defects, such as stacking faults and grain boundaries, can also prevent the spin interactions and compensate the effects of moments, which can account for the reduced remanent *Mr⊥* in the case of YbPt_Th10nm and YbPt_Th55nm (cf. Figure 7b,c). Furthermore, the lateral and vertical grain boundaries, which were revealed by TEM in Figure 6(c0) in the case of YbPt_Th55nm, have an important influence on the coercivity *Hc⊥* (see Figure 7d). Although the degree of misorientation of YbPt_Th0nm is considered high, the coercivity values *Hc⊥* were very comparable to those of YbPt_Th70nm, which indicates the reduced number of phase boundaries for YbPt_Th0nm, as could not be demonstrated by TEM in Figure 6(a0).

**Figure 8:**
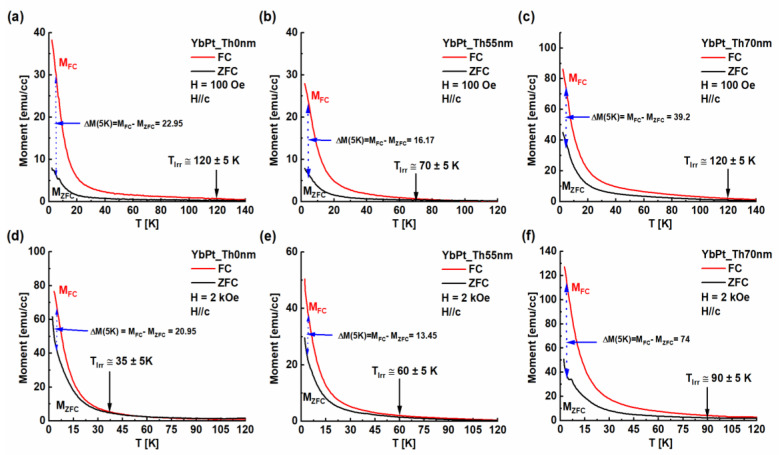
Zero-field cooling (ZFC) and field cooling (FC) curves, respectively, recorded for the YbPt_Th0nm, YbPt_Th55nm, and YbPt_Th70nm samples with an applied field *H* = 100 Oe (**a**–**c**) and H = 2000 Oe (**d**–**f**).

Temperature-dependent magnetization curves, zero-field cooling (ZFC), and field cooling (FC) were determined for YbPt_Th0nm, YbPt_Th55nm and YbPt_Th70nm, as shown in Figure 8a,d, Figure 8b,e, and Figure 8c,f, respectively. The ZFC and FC curves were recorded at two applied fields *H* = 100 Oe and 2000 Oe in the OOP direction, where the *H* was parallel to the c-axis. The thermomagnetic irreversibility (TMI) temperature (*T_Irr_*) is defined as the temperature at which the ZFC and FC curves bifurcate from each other [23]. The onset of the bifurcation temperature between the ZFC and FC curves can be considered as the magnetic ordering temperature *T_Irr_*. We introduced the bifurcation, also named the degree of irreversibility, by Δ*M(T)* between the ZFC and FC plots at a specific temperature *T*, such as Δ*M(T)* = *M_FC_* − *M_ZFC_*, and we determined Δ*M(5K)* as a benchmark for comparison purposes, as shown in Figure 8. Furthermore, the magnitude of the irreversibility Δ*M* between *M_FC_* and *M_ZFC_* was argued to be a measure of the magnetic anisotropy in the film [24]. Figure 8 shows that for *T* < *T_Irr_*, *M_FC_* continuously increased with decrease in the temperature, indicating a magnetic anisotropy in the YbPt_Th0nm, YbPt_Th55nm, and YbPt_Th70nm samples. In the opposite case, the *M_FC_* will remain constant if the anisotropy is weak in the YbFO films, which is not so in our case. It is worthwhile to point out that the total moments *M_FC_* and *M_ZFC_* have the highest values for the case of YbPt_Th70nm, which increases with the applied field *H*, indicating that the YbPt_Th70nm possesses the highest magneto-anisotropy constant, which is related to the highest crystal quality. Additionally, for YbPt_Th0nm, the magnitude of bifurcation was found to be Δ*M(5 K)* ≅ 23 and 21 at applied field *H* = 100 Oe and 2000 Oe, respectively, indicating that the irreversibility does not depend strongly on the applied field (see Figure 8a,d), since the coercivity *Hc⊥* varies between 1700 and 155 Oe for YbPt_Th0nm (see Figure 7d) in the temperature range *T* = [2 − 50 K]. For sample YbPt_Th55nm, Δ*M(5 K)* varies slightly with *H*, as illustrated in Figure 8b,e, where the *Hc⊥* varies between 612 and 4894 Oe. In contrast, the degree of irreversibility Δ*M* was stronger in the case YbPt_Th70nm and varied from Δ*M(5 K)* ≅ 39.2 to 74 as the applied field *H* was changed from 100 to 2000 Oe. This can be interpreted as a remarkable difference in the magnetic anisotropy of the sample YbPt_Th70nm in comparison with the YbPt_Th0nm and YbPt_Th55nm samples due to the interfacial difference induced by the Pt underlayer, despite the similarities recorded in the temperature-dependent *Ms⊥* and the temperature-dependent coercivity *Hc⊥*. Figure 8 shows that *T_Irr_* depends on the applied field *H* in the case of h-YbFeO_3_. Similar behavior was observed by Sahu et al. in the case of ZnFe_2_O_4_ thin films [23]. The measured *T_Irr_* ≅ 120 K at *H* = 100 Oe for samples YbPt_Th0nm and YbPt_Th70nm is in accordance with a Neel temperature *T_N_* ≅ 125 K which was recorded in former studies [4,6]. However, the measured *T_Irr_* at H = 2000 Oe was found to be *T_Irr_* ≅ 35, 60, and 90 K ± 5 K for the samples YbPt_Th0nm, YbPt_Th55nm, and YbPt_Th70nm, respectively. The transition temperature *T_Irr_* ≅ 90 K ± 5 K is lower than the value recorded by Jeong et al. [4], namely *T_N_* ≅ 120 K, for YbFO films with a thickness of 60 nm grown on Pt(111)/sapphire(0001), where Pt(111) was rather sputtered, though no information was given about the thickness of Pt bottom electrode.

Our detailed investigation revealed the influence of the structural features by the presence of the Pt underlayer on the magnetic properties depending on the temperature. In fact, achievement of a Pt underlayer of an optimum thickness improved the crystal quality of YbFO in such way that the spin interaction was not disturbed by the disturbance in the atomic arrangement as was observed in the case of Yb_Pth0nm. We conclude that the high quality of the Pt underlayer film in terms of morphology and homogeneity which was obtained in the case of Yb_PtTh70nm enabled the achievement of high magnetization at saturation *Ms⊥*, remanent magnetization *Mr⊥*, and high irreversibility temperature *T_Irr_* in comparison to the cases when *Th_Pt_* was below a value of 70 nm.

## 4. Discussion and Conclusions

The ferroelectric characterization of h-YbFeO_3_ requires the deposition of a bottom electrode, such as Pt. To the best of our knowledge, there is a lack of investigations which deal with the optimization of the Pt bottom electrode and the resulting effect of the Pt underlayer if it does not have the optimum thickness and how this could also affect not only the ferroelectric properties but also the magnetic properties, as there are few studies which confirm the multiferroicity character of h-YbFeO_3_. Our study focused on the effect of the Pt underlayer thickness on the film morphology, crystal structure, and mosaicity of h-YbFeO_3_ as well as on the magnetization parameters, such as the OOP magnetization at saturation *Ms⊥*, the OOP remanent magnetization *Mr⊥*, the coercivity *Hc⊥*, and the magnetic ordering transition temperatures *T_Irr_*. An increase in the Pt underlayer thickness *Th_Pt_* was demonstrated to improve the crystal quality of the YbFO layers. Using high-resolution transmission electron microscopy, we were able to show disturbance of the atomic arrangement in the crystal structure for the case YbPt_Th0nm, i.e., without a Pt underlayer. It is necessary to achieve an optimum *Th_Pt_* beyond 55 nm to obtain a continuous and homogenous Pt underlayer, which could improve the mosaicity of YbFO by reducing the number of phase boundaries and stacking faults. The purpose of the Pt underlayer is to reduce the misfit between the YbFO layer and the YSZ(111) substrate, to be used as a conductive bottom electrode, and not to degrade the magnetic properties in comparison with the YbFO layer deposited directly on the YSZ(111) substrate.

Our investigations demonstrate that the use of a high-quality Pt underlayer having the optimum thickness can be beneficial for the enhancement of magnetic properties. An increase in the remanent magnetization *Mr⊥* as well as in the magnitude of the bifurcation between *M_FC_* and *M_ZFC_* was achieved, which indicates the deposition of h-YbFeO_3_ with measurable magnetic anisotropy in the case of YbPt_Th70nm in comparison to YbPt_Th0nm for comparable deposited YbFO layer thicknesses.

## Figures and Tables

**Table 1:** Summary of the main parameters derived from X-ray diffraction and microscopy analysis methods, organized in the following sections: 1. PLD-growth conditions; 2. Film characterization; 3. Film morphology. NA: Non-applicable.

1. PLD Growth Conditions	YbPt_Th0nm	YbPt_Th10nm	YbPt_Th40nm	YbPt_Th55nm	YbPt_Th70nm
Number of shots *N^YbFO^sh*	20,000	20,000	20,000	20,000	20,000
Growth temperature *T^YbFO^g* [°C]	900	900	900	900	900
Number of shots *N^Pt^sh*	NA	1647	8235	12,350	16,470
Growth temperature *T^Pt^g* [°C]	NA	900	900	900	900
**2. Film Characterization**	**YbPt_Th0nm**	**YbPt_Th10nm**	**YbPt_Th40nm**	**YbPt_Th55nm**	**YbPt_Th70nm**
*Th_YbFO_* (XRR) [nm]	95 ± 5 nm	128.61 ± 5 nm	125.8 ± 5 nm	105.6 ± 5 nm	120 ± 5 nm
*Th_Pt_* (Mass density) [nm]	NA	13.67 ± 5 nm	49.08 ± 5 nm	50.87 ± 5 nm	80.52 ± 5 nm
*Th_Pt_* (TEM) [min-max] <*Th_Pt_* (TEM)> [nm]	NA	NA	[18–96] 33.6 ± 5	[50–68] 56.5 ± 5	[64.6–86.15] 75.16 ± 5
*Th_YbFO_* (TEM) [min-max] <*Th_YbFO_* (TEM)> [nm]	NA 94 ± 5	NA	[108–120] 114 ± 5	[103–117] 110 ± 5	NA 95 ± 5
**3. Film Morphology**	**YbPt_Th0nm**	**YbPt_Th10nm**	**YbPt_Th40nm**	**YbPt_Th55nm**	**YbPt_Th70nm**
Degree of coverage [%]					
Yb_DoC (EDX) [%]	NA	86.36 ± 2	92.62 ± 2	93.83 ± 2	NA
DoC (BSE) [%]	100 ± 2	NA	95.34 ± 2	94.33 ± 2	NA

**Table 2:** Summary of the main parameters derived from X-ray diffraction and microscopy analysis methods, organized in the following sections: 4. Crystal structure and 5. Mosaicity. NA: Non-applicable.

4. Crystal Structure	YbPt_Th0nm	YbPt_Th10nm	YbPt_Th40nm	YbPt_Th55nm	YbPt_Th70nm
dspacing of YSZ ${d(11\bar{2})}_{YSZ}$ [Å]	2.0985 ± 0.0005	2.1002 ± 0.0005	2.1004 ± 0.0005	2.1004 ± 0.0005	2.1004 ± 0.0005
dspacing of Pt ${d(11\bar{2})}_{Pt}$ [Å]	NA	1.60280 ± 0.0005	1.6023 ± 0.0005	1.5944 ± 0.0005	1.5977 ± 0.0005
In-plane lattice parameter *a_YbFO_* [Å]	5.9603 ± 0.0005	5.8685 ± 0.0005	5.8750 ± 0.0005	5.9167 ± 0.0005	5.9544 ± 0.0005
Out-of-plane lattice parameter *c_YbFO_* [Å]	11.7086 ± 0.0005	11.7292 ± 0.0005	11.7192 ± 0.0005	11.7087 ± 0.0005	11.6990 ± 0.0005
Misfit *f_YbFO/YSZ_* [%]	−5.32 Compressive	NA	NA	NA	NA
Misfit *f_YbFO/Pt_* [%] (Yb/4 × Pt)	NA	−8.46 Compressive	−8.34 Compressive	−7.22 Compressive	−6.83 Compressive
Misfit *f_Pt/YSZ_* [%]	NA	−23.07 Compressive	−23.16 Compressive	−22.92 Compressive	−23.37 Compressive
In-plane residual strain *ε^//^_YbFO_* [%]	−0.0115 ± 0.001	−0.0268 ± 0.001	−0.0257 ± 0.001	−0.0188 ± 0.001	−0.0126 ± 0.001
Out-of-plane plane residual strain *ε^⊥^_YbFO_* [%]	0.0025 ± 0.0002	0.0042 ± 0.0002	0.0034 ± 0.0002	0.0025 ± 0.0002	0.0016 ± 0.0002
**5. Mosaicity**	**YbPt_Th0nm**	**YbPt_Th10nm**	**YbPt_Th40nm**	**YbPt_Th55nm**	**YbPt_Th70nm**
Degree of misorientation *α_YbFO_* [degree]	0.93 ± 0.05	0.99 ± 0.05	1.07 ± 0.05	0.8 ± 0.05	0.48 ± 0.05
Lateral size mosaic blocks *L^H^_YbFO_* [nm]	NA	179.0 ± 5	218.2 ± 5	NA	571.2 ± 5
Vertical size mosaic blocks *L^V^_YbFO_* [nm] (2xFWHM)	81.8 ± 5	39.35 ± 5	60.55 ± 5	52.7 ± 5	100.35 ± 5
Mean value vertical strain distribution *<β^⊥^_YbFO_>* (2xFWHM)	3.95 × 10^−3^ ± 0.5 × 10^−4^	2.80 × 10^−3^ ± 0.5 × 10^−4^	3.58 × 10^−3^ ± 0.5 × 10^−4^	2.40 × 10^−3^ ± 0.5 × 10^−4^	1.82 × 10^−3^ ± 0.5 × 10^−4^

## Data Availability

The data presented in this study are available on request from the corresponding author.
